# The Influence of Online Social Value Co-creation Activity on Consumer Purchase Intention: An Experimental Study

**DOI:** 10.3389/fpsyg.2022.951891

**Published:** 2022-06-29

**Authors:** Ying Shi, Jinjin Zheng, Mo Liang

**Affiliations:** ^1^College of Vehicle Engineering, Changzhou Vocational Institute of Mechatronic Technology, Changzhou, China; ^2^School of Management, Shanghai University, Shanghai, China; ^3^USC-SJTU Institute of Cultural and Creative Industry (ICCI), Shanghai Jiao Tong University, Shanghai, China

**Keywords:** social value co-creation, consumer-company identification, social norms, social interaction theory, experiments

## Abstract

In recent years, an increasing number of online social value co-creation activities are conducted by companies in their marketing campaigns. A question is that whether these activities that take social responsibilities could help enterprises improve marketing performance. Drawing from social interaction theory, this study explores the causal effect of online social value co-creation on consumer purchase intention through three experiments. The results show that social value co-creation can stimulate consumer purchase intention. Moreover, consumer-company identification plays a mediating role in linking social value co-creation to purchase intention. In addition, compared to low social norms, high social norms are more likely to weaken the influence of social value co-creation on consumers' buying intention. The study provides both theoretical and practical implications to research area. Limitation and future research directions are also discussed.

## Introduction

In recent years, digital economy brought great changes to all the aspects in our society, including consumption. Marketing styles and consumer behavior are substantially affected by the Internet and digital technology (Shapiro et al., 1998; Stiglitz, 2002). The development of the digital economy helps enterprises acceleratingly launch the diversified online value co-creation activities. For instance, an increasing number of enterprises involve consumers in their online value creation activities, through which they establish connections with consumers (Rodell et al., 2020). Different from traditional value co-creation, social value co-creation is a novel marketing strategy for enterprises to build close contacts with consumers by launching the activities that emphasize social responsibility (Prahalad and Ramaswamy, 2004; Vargo and Lusch, 2008; Jurietti et al., 2017). For instance, the Ant Forest project run by Alibaba—one of the largest Internet companies in China incorporates public donations and agricultural assistance activities that aim at alleviating public poverty and enhancing social welfare. However, it is still not clear that how online social value co-creation activities influence consumers purchase intentions and the mechanism by which they act.

When reviewing the existing literature, we find the following limitations. Firstly, most previous studies on value co-creation are from the perspective of enterprises or consumers (e.g., Morsing and Schultz, 2006; Korschun and Du, 2013; Agrawal et al., 2015). Few studies pay attention to the social value in the co-creation activities which is increasingly important in the contemporary society. Thus, it is necessary to investigate the features of social value co-creation activities in order to enrich the relevant theory. Secondly, the existing studies mainly focus on the antecedents of consumers' participation in social value co-creation activities, but fail to explore its consequences. Consumers purchase intention is one of the key outcomes of marketing activities. This research explores how social value co-creation activities affect consumer purchase intention. In addition, previous studies assert that organizations can be a key component of individuals' social identity (Brewer, 1991). Consumer identification with a company can be constructed through social value co-creation activities and in result affects consumers' purchase intention (Bhattacharya and Sen, 2003). In this study we examines the role of consumer-company identification in promoting purchase intention in social value co-creation activities. Moreover, social influence always exerts great impacts on consumer decision-making process (e.g., Rook and Fisher, 1995; Kuan et al., 2014). Therefore, we expend the research scope by taking social norms as the boundary condition that moderates the effect of social value co-creation on consumer purchase intention. Lastly, most of the relevant studies that use cross-sectional data fail to achieve causality. In the current study, we adopt three scenario experiments to explore the mechanism by which online social value co-creation activities enhance consumer purchase intention, including the direct, mediating and moderating effects. Theoretical and practical implications are also provided.

## Theory and Hypothesis

### Social Value Co-creation and Consumer Purchase Intention

Social interaction theory maintains that the interaction between enterprises and consumers is an efficient way to co-create value for both (Prahalad and Ramaswamy, 2004; Jurietti et al., 2017). Thus, value co-creation activities can enhance consumer purchase intention (Payne et al., 2008; See-To and Ho, 2014). Social co-creation activities are different from traditional value co-creation ones in emphasizing social welfare contribution to the society. Recently, growing attention has been paid to corporate social responsibility (CSR) issues. A great number of studies stress the importance of CSR as a competitive advantage through analyzing its positive consequences on company performance. For instance, Du et al. (2010) summarize that companies that engage in CSR activities can generate favorable stakeholder attitudes and behaviors. Moreover, it is evidenced that consumers' perceptions of an enterprise's CSR enhance purchase intention toward its product or service (e.g., David et al., 2005; Lee and Shin, 2010; Mulaessa and Wang, 2017). Chu and Chen (2019) find that consumers' participation in CSR-related activities in social media promotes purchase intention through enhancing identification with the brand and positive brand attitude. Zhang and Ahmad (2021) discover that CSR affects consumers' purchase attention through trust and brand image.

Thus, companies tend to encourage consumers to actively participate in the activities that stress social value creation (Prahalad and Ramaswamy, 2004; Jurietti et al., 2017). During the participation, consumers can feel a sense of social responsibility accomplishment, and then obtain more satisfaction. At the meantime, consumers may intuitively appreciate the enterprises' efforts for social welfare. Companies can also achieve economic returns through engaging in prosocial initiatives (Rodell et al., 2020). Thus, we postulate consumers' participation in social value co-creation activities will enhance their purchase intention.

H1: Online social value co-creation activities enhance consumer purchase intention.

### The Mediating Role of Consumer-Company Identification

Consumer identification with a company is defined as “an active, selective, and volitional act motivated by the satisfaction of one or more self-definitional needs” (Bhattacharya and Sen, 2003, p. 77). Social identity theory maintains that people tend to develop a social identity by categorizing themselves as members of various social groups such as gender, occupation, ethnicity, as well as organization (Brewer, 1991). Some researchers even maintain that organizations are the key component of people's social identity (Bhattacharya and Sen, 2003). The concept was initially studied from the perspective of employees in the field of management (e.g., Mael and Ashforth, 1992; Pratt, 1998). Management researchers discover that person-organization identification can help employees build trust in organizations (Kramer, 1999) and influence some work outcomes such as affective commitment (Bergami and Bagozzi, 2000). In addition, individuals may have a sense of identification with an organization even they are not its formal members (Scott and Lane, 2000). Then marketing scholars started applying the concept in consumer research. Bhattacharya and Sen (2003) maintain that consumer identification with a company can help build a close consumer-company relationship that satisfy consumers' key self-definitional needs. Such consumer-company identification motivates consumers to engage in company-related issues.

Previous studies propose that consumers' CSR perceptions strengthen consumer-company identification through brand attractiveness (Currás-Pérez et al., 2009). Specifically, when consumers participate in social value co-creation activities, a positive image toward the enterprise will be generated, and then stimulates consumer-company identification to satisfy consumers' self-definitional needs. Furthermore, the more individuals identify with an organization, the more likely they are to purchase the products provided by the company (Currás-Pérez et al., 2009). Thus, we propose a link between social value co-creation activities, consumer-company identification and purchase intention. Specifically, consumer identification with a company may play a mediating role in the relationship between social value co-creation activities and consumer purchase intention.

H2: Consumer-company identification mediates the effect of online social value co-creation activities on consumer purchase intention.

### The Moderating Effect of Social Norms

Social norms can regulate individual behaviors during social interactions (Cialdini and Goldstein, 2004). Social norms include imperative and descriptive norms. Descriptive norms are informal and mandatory, while imperative norms are formal mandatory. Marketing researchers mainly focus on the impact of the former one on consumer behavior (e.g., Rook and Fisher, 1995; Kuan et al., 2014). The normative influence is not only from family members and close friends, but also from distant friends and colleagues (Toker-Yildiz et al., 2017). Rook and Fisher (1995) find consumers' normative evaluations moderate the relationship between the buying impulsiveness trait and consumers' buying behaviors. In E-commerce field, Kuan et al. (2014) explore the role of social influence affects online group-buying behavior. In social value co-creation activities, consumers are also inevitably affected by social groups during the process of interpersonal interactions. In the high social norm scenarios with social value co-creation (vs. low social norm scenarios), individuals are more likely to make purchase decisions to gain social approval. However, besides extrinsic peer pressure, consumers are greatly motivated by intrinsic social responsibility in social value creation buys. When they are informed that people around them also buy these products, they may feel it is not that urgent to make such purchases. Instead, they tend to spend money on those products with social value but lack of attention. They believe purchasing such items can make greater contribution to the society. Therefore, we postulate that under low social norms, the effect of social value cocreation on purchase intentions is stronger than that under high social norms.

H3: Social norms enhance consumer purchase intention.

H4: Social norms moderate the positive effect of social value co-creation on consumer purchase intention, such that it will be stronger when the social norms at low level rather than at high level.

## Experiment 1

The main purpose of this experiment was to assess the validity of the materials and explore the effect of social value co-creation (vs. the control condition) on consumer purchase intentions.

### Experimental Design and Measures

A two-group (social value co-creation vs. the control condition) between-subject experiment was adopted to test H1. Participant were recruited in an e-commerce forum. Specifically, the researchers attach the experiment web link in the forum and data were collected through an online survey platform. Sixty-seven valid samples were included in this study, which incorporated 38 females (56.7%) and 29 males (43.3%) with a mean age of 27.48. Participants were randomly assigned to one of the groups. There were 33 in the social value co-creation group and 34 in the control group, respectively.

The experimental materials were made based on prior studies on the manipulation of social value co-creation (e.g., Deng and Xu, 2017). The participants were required to watch the scenario video that depicts how purchasing agricultural products helps farmers in need. Then they were informed that when browsing e-commerce page, they find some unsalable agricultural products they like. In control condition, the participants were only told that when browsing e-commerce website, they find some agricultural products they are interested in.

After reading the material, participants responded to the scales that measure social value co-creation and purchase intention. Seven items were adapted from Tang and Jiang (2018) and Deng and Xu's (2017) to measure social value co-creation perception. Three items from Lu et al. (2010) were adopted to measure purchase intention. All of items were rated on 5-point Likert scales (1 = strongly disagree and 5 = strongly agree). Finally, participants were required to report their gender, age, and education background.

### Results

The Cronbach's alpha coefficients for social value co-creation and purchase intention were 0.76 and 0.77, respectively, demonstrating the scales have sound reliabilities. The mean score for social value co-creation perception was significantly higher (*t* = 6.33, *p* < 0.001) in the social value co-creation scenario (M = 4.32, SD = 0.40) than that in the control condition scenario (M = 3.64, SD = 0.47), indicating the manipulation was successful.

**Figure 1:**
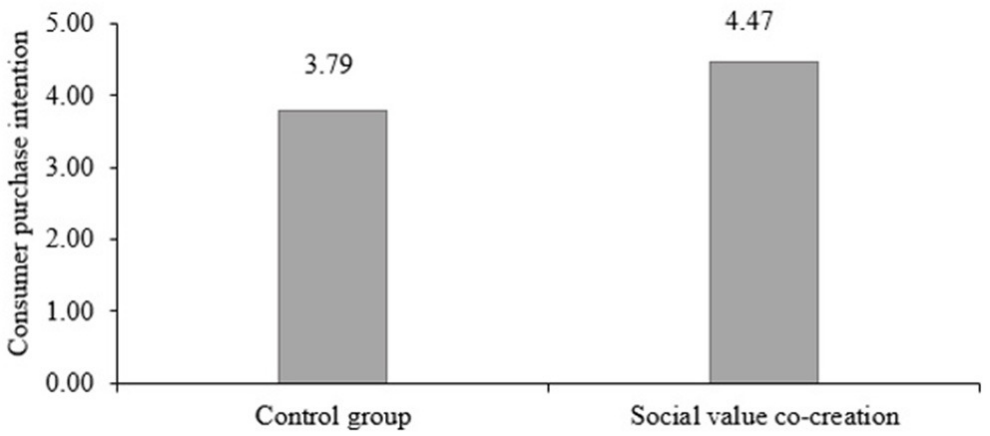
The effect of social value co-creation (vs. control group) on consumer purchase intention.

To test H1, we performed a *t*-test to compare consumer purchase intentions between the two groups. The result in Figure 1 demonstrates the participants in the social value co-creation activity reported significantly higher purchase intention (M = 4.47, SD = 0.47; *t* = 5.58, *p* < 0.001) than their counterparts in the control condition (M = 3.79, SD = 0.52), which verifies H1.

## Experiment 2

Experiment 2 was conducted to further examine the mediating effect of consumer-company identification in the relationship between social value co-creation and purchase intention.

### Experiment Design and Measures

A two-level (the social value co-creation group vs. the control group) between-subject experiment was adopted to examine H2. Data collection method is the same as Experiment 1. The experiment includes 64 participants with the average age of 23.36. Of the participants, 54.7% were male and 45.3% were female. The participants were randomly assigned to one of the experiment conditions. There were 33 in the social value co-creation group and 31 in the control group, respectively.

After watching the same video as that in Experiment 1, they are required to respond to a questionnaire with three scales—perceived social value co-creation, purchase intention and consumer identity. Consumer-company identification was measured by Kang et al. (2015) 3-item scale. All the items were using 5-point Likert scales (1= strongly disagree and 5= strongly agree). Gender, age, and education background were also provided by the participants.

### Data Analysis and Results

The Cronbach's alpha coefficients for social value co-creation, purchase intention and consumer-company identification were 0.88, 0.78, and 0.83 respectively, indicating that all the scales had sound reliability. Then we conduct manipulation check. The result shows that the participants in social value co-creation group had significantly higher social value co-creation perception (M = 4.27, SD = 0.36; *t* = 8.69, *p* < 0.001) than their control group counterparts (M = 3.32, SD = 0.49). Moreover, their purchase intention (M = 4.24, SD = 0.44; *t* = 6.86, *p* < 0.001) is also significantly higher than the participants in the control group (M = 3.43, SD = 0.50), supporting H1 again.

We further tested the mediating effect of consumer-company identification using the PROCESS macro with SPSS. The results reflect that the confidence intervals for the indirect effect of social value co-creation on purchase intention through consumer-company identification excludes the value of 0 [95% CI = (0.07, 0.49)]. The mediating effect is 0.66. After controlling consumer-company identification, the direct influence of social value co-creation on purchase intention is significant [95% CI = (0.33, 0.78), excluding 0]. Thus, H2 was supported.

## Experiment 3

### Experiment Design and Measures

Experiment 3 was designed to further test the interactive effect of social norms and social value co-creation on consumer purchase intention. Specifically, a 2 (the social value co-creation group vs. the control group) × 2 (high vs. low social norms) between-subject experiment was conducted. We still used the same data collection methods as the previous experiments. The participants were randomly assigned to one of the four scenario conditions. Finally, 142 valid respondents were included. Of the samples, 82 were females (57.7%) and 60 were males (42.3%) with the mean age of 23.45. There were 35 in the high social norms and social value co-creation group, 34 in the low social norms and social value co-creation group, 36 in the high social norms and control group, and 37 in the low social norms and control group.

We manipulated social norms through letting the participants read the scenario materials. In high social norms group, participants were informed that most of their colleagues, friends and family members are concerned about and tend to purchase the agricultural products in the previous video. In low social norms group, participants were told that few individuals around them care about those products.

Subsequently, the participants respond to the scales of social value co-creation perception, consumer-company identification, social norms perception and purchase intention scales. Social norms perception is measured by five items we made specifically for the current research. A sample item is that “People around me are interested in these products”. All scales were assessed using 5-point Likert scales response format (1 = strongly disagree and 5 = strongly agree). Finally, the participants were required to provide their gender, age, and educational background.

### Data Analysis and Results

The Cronbach's alpha coefficients for social value co-creation, consumer-company identification, social norms and purchase intention were 0.84, 0.82, 0.92, and 0.81, respectively, indicating that all the scales had good reliability.

In manipulation check, the mean score of social value co-creation perception for the manipulation group (M = 4.03, SD = 0.50; *t* = 9.45, *p* < 0.001) is again significantly higher than the control group (M = 3.14, SD = 0.62). Moreover, the mean score of social norms perception in the high social norms group (M = 4.09, SD = 0.61; *t* = 7.39, *p* < 0.001) is significantly higher than that in the low social norms group (M = 3.02, SD = 1.05). The result demonstrates that the manipulations of both social value co-creation and social norms are successful.

Then we tested the main effect. The *t*-test result reveals that the participants who joined in the social value co-creation scenario (M = 4.12, SD = 0.60; *t* = 7.62, *p* < 0.001) have significantly higher purchase intentions than those in the control group (M = 3.17, SD = 0.85). The main effect of social value co-creation was verified. Furthermore, we tested the mediating effect by using the PROCESS macro with SPSS. The results shows that the confidence intervals for the indirect effect of social value co-creation on consumer purchase intention through consumer-company identification excluded the value of 0 [95% CI = (0.49, 0.83)]. The mediating effect is 0.66. After controlling the mediator, the direct effect of social value co-creation on purchase intention is significant (LLCI = 0.02, ULCI = 0.31, excluding 0), demonstrating a partial mediating effect of consumer-company identification. Thus, H2 was supported again.

Next we examined the role of social norms in social value co-creation activities. We firstly adopted a *t*-test to test the main effect of social norms. Under the high social norms condition, the mean score of purchase intention (M = 3.91, SD = 0.68; *t* = 3.63, *p* < 0.001) is significantly higher than that under low social norms condition (M = 3.40, SD = 0.96). The result demonstrates social norms can enhance consumer purchase intention in both contexts (the social value co-creation condition and the control condition), supporting H3. Then we perform a two-way ANOVA to test the interactive effect of social value co-creation and social norms on consumer purchase intention. The result showed that the main effects of both social value co-creation (F = 72.69, *p* < 0.01) and social norms (F = 23.49, *p* < 0.01) on purchase intention are significant. Moreover, their interactive effect on purchase intention is also significant (F = 4.05, *P* < 0.05).

**Figure 2:**
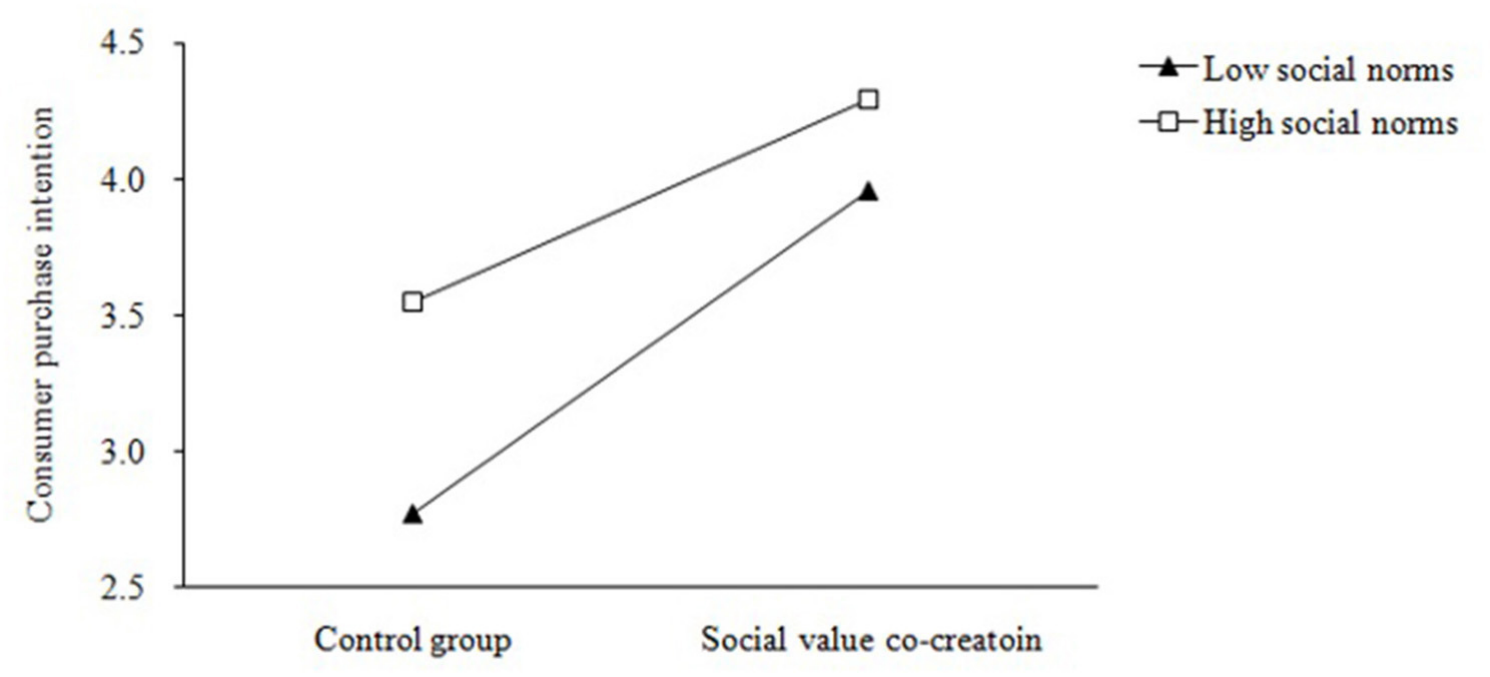
The interaction effect of social value co-creation on purchase intention.

Specifically, under high social norms, consumer purchase intention for the social value co-creation group (M = 4.29) is higher than the control group (M = 3.55); while under low social norms, the mean score of purchase intention is 3.96 for social value co-creation group, significantly higher than that of the control group (M = 2.77). Figure 2 illustrates that the effect of social value co-creation on purchase intention is stronger when social norms are high than low. Therefore, H4 was verified.

## Discussion

In the rapid development of the digital economy, a growing number of enterprises invite their customers to launch activities for social value co-creation activities through which they closely establish relations with consumers to realize corporate goals. Drawing from social interaction theory, we explore the effects of value co-creation, consumer-company identification and social norms on purchase intention. Specifically, the results reveal that social value co-creation can enhance consumer purchase intention though consumer-company identification. Moreover, the effect is moderated by social norms.

### Theoretical Implications

As one of the few experimental studies in the area, we examine the causal relationships between the variables investigated and depict a comprehensive picture of consumer decision-making process in social value co-creation activities. This study extends the understanding of consumers' social value co-creation engagement and examines the mechanism of its influence on purchase behavior. Through the scenario experiments, we find the participation in social value co-creation activities can help enhance consumers' purchase intention toward specific products through the increase of consumers' identification with the company. Moreover, social norms are identified as a moderator that can mitigate the effect of social value co-creation on purchase intention. This result further demonstrates the role of social norms in consumer behavior and psychology.

### Practical Implications

With the development of the digital economy, it is more convenient for enterprises to incorporate the consumers in the social value co-creation activities. Thus, our research offers practical implications for the practitioners.

First, the enterprises should attach more importance to social value co-creation with consumers. In one hand, it can stimulate the consumers' purchase intentions through enhancing consumers' identification with the companies. Moreover, it motivates enterprises to take more social responsibility which is beneficial to the whole society. Second, the companies should organize various social value co-creation activities to avoid the formation of social norms that let consumers feel most others around them are taking part in specific social value co-creation activities, which as a result suppress the effect of these activities on consumers' buying intentions. Last, the government should encourage companies to launch social value co-creation activities that benefit all of the stakeholders.

### Limitations and Future Research

Although this study provides some insightful implications from both theoretical and practical perspectives, there are still some limitations that should be further addressed in the future research. First, our study responds to the call in previous studies to pay attention to the consequences of social value co-creation. There are still some other outcomes of social value co-creation that deserve to be investigated, such as consumer satisfaction and consumer wellbeings. Moreover, besides consumer-company identification, exploring other mediators that can link social value co-creation to consumer purchase intention is highly expected.

Secondly, we only focus on unsalable agricultural products in the current research. In the future the scholars should examine the consequences of various types of social value co-creation activities. Finally, the respondents in this study are relatively young. This might be because we conduct the experiments through online platform. Future studies should extend the sample to other generation cohorts in order to extend the generalizability of the findings in this study.

## Data Availability Statement

The raw data supporting the conclusions of this article will be made available by the authors, without undue reservation.

## Ethics Statement

Ethical review and approval was not required for the study on human participants in accordance with the local legislation and institutional requirements. Written informed consent for participation was not required for this study in accordance with the national legislation and the institutional requirements.

## Author Contributions

YS and JZ developed the theoretical framework and completed manuscript writing. JZ and ML worked on experiment design and data collection and analysis. All authors contributed to the article and approved the submitted version.

## Conflict of Interest

The authors declare that the research was conducted in the absence of any commercial or financial relationships that could be construed as a potential conflict of interest.

## Publisher's Note

All claims expressed in this article are solely those of the authors and do not necessarily represent those of their affiliated organizations, or those of the publisher, the editors and the reviewers. Any product that may be evaluated in this article, or claim that may be made by its manufacturer, is not guaranteed or endorsed by the publisher.
